# Challenges in Solvent-Free Methods for Manufacturing Electrodes and Electrolytes for Lithium-Based Batteries

**DOI:** 10.3390/polym13030323

**Published:** 2021-01-20

**Authors:** Nina Verdier, Gabrielle Foran, David Lepage, Arnaud Prébé, David Aymé-Perrot, Mickaël Dollé

**Affiliations:** 1 Département de Chimie, Université de Montréal, CP6128 Succursale Centre-Ville, Montréal, QC H3T 1J4, Canada; nina.verdier@umontreal.ca (N.V.); gabrielle.foran@umontreal.ca (G.F.); david.lepage.3@umontreal.ca (D.L.); arnaud.prebe@umontreal.ca (A.P.); 2 Total SA, 2 Pl. Millier, 92069 Paris La Défense, France; david.aymeperrot@total.com

**Keywords:** solvent-free processes, solid polymer electrolyte, electrodes, polymer binder, melt-processing, hot-pressing, Li-ion batteries, Li metal batteries

## Abstract

With the ever-growing energy storage notably due to the electric vehicle market expansion and stationary applications, one of the challenges of lithium batteries lies in the cost and environmental impacts of their manufacture. The main process employed is the solvent-casting method, based on a slurry casted onto a current collector. The disadvantages of this technique include the use of toxic and costly solvents as well as significant quantity of energy required for solvent evaporation and recycling. A solvent-free manufacturing method would represent significant progress in the development of cost-effective and environmentally friendly lithium-ion and lithium metal batteries. This review provides an overview of solvent-free processes used to make solid polymer electrolytes and composite electrodes. Two methods can be described: heat-based (hot-pressing, melt processing, dissolution into melted polymer, the incorporation of melted polymer into particles) and spray-based (electrospray deposition or high-pressure deposition). Heat-based processes are used for solid electrolyte and electrode manufacturing, while spray-based processes are only used for electrode processing. Amongst these techniques, hot-pressing and melt processing were revealed to be the most used alternatives for both polymer-based electrolytes and electrodes. These two techniques are versatile and can be used in the processing of fillers with a wide range of morphologies and loadings.

## 1. Introduction

Li-ion batteries are a well-established and mature technology that has spread to almost, if not all, of our portable devices. Li-ion batteries are composed of a positive and a negative electrode as well as an electrolyte to ensure the transfer of lithium ions through the cell. If the electrolyte is liquid, a membrane called separator is used to avoid contact between the two electrodes. Electrodes are then connected thanks to an external circuit allowing electrons to go from one electrode to the other. Li-ion batteries are based on the insertion/deintercalation of lithium ions inside the electrode, more specifically into inorganic materials commonly called active materials due to their role inside electrodes. Such batteries have conquered the battery market as they present several advantages compared to other battery types like alkaline or lead-acid batteries. Li-ion batteries have higher specific power and specific energy and show an increased cycle life [1]. For these reasons, this technology is one of the most promising for use in the developing electric vehicle (EV) market. Mostly due to the high demand in the automotive industry for EVs, the fabrication of Li-ion batteries has drastically increased and is predicted to rise even more in coming years. The creation of batteries requires the formulation and manufacturing of an electrolyte and electrodes, the latter being made via the solvent-casting method. For an electrode, this classical process is based on pre-dissolving a polymer into an organic, usually toxic, solvent. The different particles, which are the conductive carbons and the active material are then added in order to make a slurry. This slurry is casted onto a current collector thanks to the doctor-blade technic. A good representation of this technic can be seen in Marks et al.’s publication [2]. The final step to obtain a solid electrode is to evaporate the remaining solvent, usually thanks to heat and vacuum. The increasing production of batteries therefore raises the issue of using more environmentally friendly and cheaper processing methods that do not require the use of solvents.

Another concern regarding the lithium batteries are the safety issues related to the use of liquid electrolytes. The organic solvents needed to make these electrolytes are highly flammable and, when used with Li metal anodes, can result in the formation of Li dendrites which can cause the device to short-circuit. To circumvent these constraints, solid electrolytes can be used. Promising solid electrolytes include solid polymer electrolytes (SPE), ceramics [3], and hybrid polymer electrolytes that contain inorganic fillers [4]. The use of flexible polymer-based electrolytes leads to improved surface contact at the interface with the electrode which can be an issue with brittle ceramic systems. Polymer-based electrolytes can also act as separators decreasing the battery’s weight and will mostly allow the use of Li metal as the anode. Device safety also increases because no flammable organic solvent is needed and the mechanical strength of the polymer-based electrolyte is generally sufficient to act as a physical barrier to limit dendrite growth [5]. Polymer electrolytes and hybrid polymer electrolytes are now under intense investigation to find the most efficient component which could replace definitively liquid electrolytes in commercial Li-ion batteries.

Hence, research on all-solid-state batteries is very active due to the high potential of these devices to be the batteries of tomorrow. Although the race has been centered on creating new high-performance polymer-based electrolytes or electrodes, the other concern in the community is the processes that are used to make these components. Though the solvent-casting method is the most common process for making porous and non-porous electrodes as well as polymer-based electrolytes [6,7,8], several groups have been working on new processes to replace the use of solvents. The solvent-casting technique raises the potential issues of a time consuming drying step [7] and the presence of residual solvent [9,10,11] which can be detrimental to battery performance and safety (presence of water with Li metal or coordination of water at the surface of ceramics that are in the hybrid electrolyte [12]). Moreover, this process is limited to making thick electrodes and the long drying time can lead to the agglomeration/sedimentation of particles resulting in non-homogenous samples, decreasing the performance of the electrolyte or the electrode [13]. Also, solvent evaporation leaves pores which are unnecessary in solid electrolytes and electrodes used in all-solid-state batteries [14]. Solvent-free preparation of polymer-based electrolytes and electrodes has emerged as a response to these limitations.

**Figure 1:**
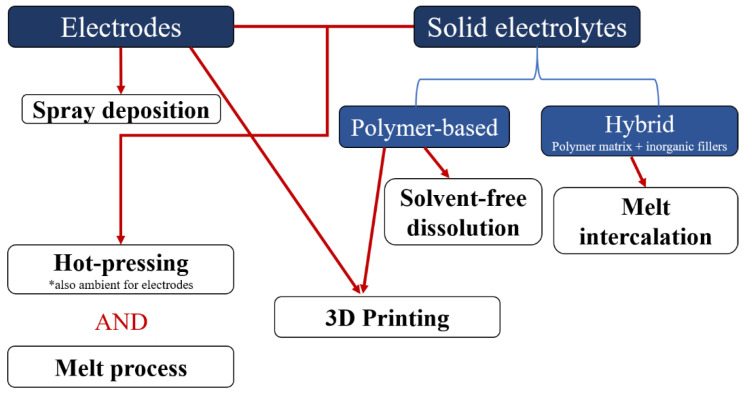
Flow chart summarizing the different solvent-free processes recently developed to make electrodes and/or solid electrolytes for lithium-ion batteries.

This review reports on different solvent-free processes that have been developed to make polymer-based electrolytes and electrodes (non-porous and porous) for Li-ion and Li-metal batteries. The different processes mentioned in this review are summarized in Figure 1. The first part of this review focuses on processes that are used to make solid polymer electrolytes (polymer and salt) and hybrid polymer electrolytes (polymer, salt and ionically conductive or non-conductive fillers), both being promising alternatives for replacing classical liquid organic electrolytes. The second part of this review emphasizes electrode manufacturing with an overview of processes that can be used at high particle loadings. Both porous and non-porous electrodes will be discussed in this part.

## 2. Dry Processes for Polymer Electrolytes

### 2.1. Solid Polymer Electrolytes (SPE)

Since the first studies conducted by Wright [6] and Armand et al. [15] in the late 1970s, solid polymer electrolytes have attracted a lot of attention. These materials are of primary interest as they increase the safety of Li-ion batteries as the polymers are less flammable than the organic solvents that are used in classical liquid electrolytes. Moreover, the increased mechanical strength of solid polymer electrolytes can play a role in diminishing dendrite growth allowing Li metal to be used as an anode [5,16].

Since their discovery, the main process for creating solid polymer electrolytes is to dissolve the polymer and the salt into a volatile organic solvent, often acetonitrile, and then cast the solution to obtain a polymeric film by solvent evaporation. This approach has several drawbacks including that the remaining traces of solvent affect the conductivity of the resultant electrolyte [17,18], the use of toxic solvents and their costs, the drying time which can result in phase separation of the SPE or salt crystallization and, finally, the equipment that is necessary to trap the vapors (industrial condenser) during the evaporation step is expensive. To circumvent these limitations, other manufacturing processes have been proposed and will be described in detail here.

#### 2.1.1. Solvent-Free Dissolution

The idea of preparing SPE without solvents seems to have been present since the early days of SPE manufacture. A potential solvent-free method that has come to light from these early days is dissolving the salt directly into a molten polymer or into low molecular weight polymers which are liquid at ambient temperature.

For example, Watanabe et al. published a study in 1984, in which a SPE was made by the direct dissolution of the salt into the molten polymer at high temperature. They tested this process with poly (ethylene succinate) (PES) and LiClO_4_. The polymer and the salt were mixed at ambient temperature and heated at 120 °C (T_m_(PES) is around 105 °C) under a nitrogen stream. After the complete dissolution of LiClO_4_ into the molten polymer, the blend was cooled to room temperature and ground. The above procedure was repeated twice to ensure sample homogeneity. Finally, the film was annealed at 60 °C under vacuum for 24 h to reach equilibrium crystallization of the polymer [19]. Even if this technique has the advantage of being solvent-free, it is still time consuming as the heating and cooling step needs to be repeated several times. These steps can be avoided by using a polymer that is liquid at ambient temperature such as low molecular weight poly (ethylene glycol) (PEG). Wang et al. used this approach to synthesize a star-comb copolymer to form their SPE. They took advantage of the liquid poly (ethylene glycol) methacrylate (PEGMA) and dissolved vinyl-functionalized poly(L-lactide) (PLLA) and LiTFSI into it. The resultant solution was mixed until homogenous. A photo-initiator was then added to the solution, which was poured into a stainless steel substrate in order to UV cross-link the blend to produce a self-standing SPE membrane [20].

The solvent-free dissolution of salt into a molten polymer or into a low molecular weight liquid polymer is an interesting alternative to avoid the use of solvents. The drawback of this technique is that it is limited to polymers that can be processed in the molten state and those that are liquid under ambient conditions. Moreover, the salt needs to be soluble in the molten or liquid polymer, which is not always the case.

#### 2.1.2. Hot-Pressing

**Figure 2:**
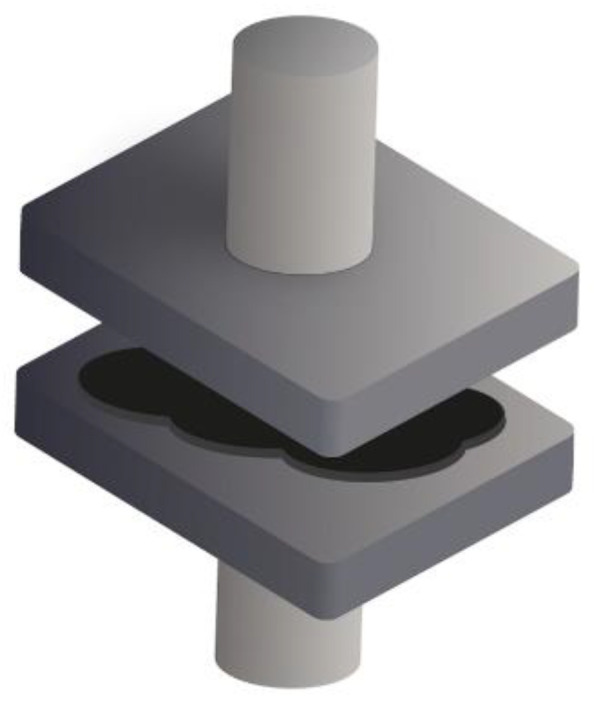
Preparation of a solid polymer electrolyte by the hot-pressing method. High temperature and high pressure are required to shape the membrane.

The hot-pressing method has also been used since the early stages of SPE development. It involves mixing the polymer and the salt at ambient temperature, often in powder form, and then pressing the mixture under high temperature and pressure (Figure 2).

For this approach, the temperature and the pressure applied need to be chosen based on the polymer that is used. For example, MacFarlane et al. formed a SPE composed of polyvinyl alcohol (PVA) and LiCF_3_SO_3_ (T_m_ (PVA) is around 160–170 °C) via the hot pressing method by heating the sample at 110 °C for 2 to 4 h, depending on the salt content, with a pressure of 7 tons [21]. Forsyth et al. prepared polyacrylonitrile (PAN)-LiCF_3_SO_3_ SPE by pressing the mixture around 150 °C with a pressure of 5–7 tons, but the time required to obtain an homogenous film was not documented [22]. In both cases, it needs to be highlighted that the processing temperature must be lower than the T_m_ of the polymer as high pressures help the polymer to flow. However, the powder pre-mixing conditions, the powder size, the granulometric dispersion, and the pressing conditions all have a significant impact on the quality of the SPE’s homogeneity. To circumvent this limitation, Kim et al. added room temperature ionic liquids (RTILs) to poly (ethylene oxide) (PEO) and LiTFSI. RTILs can be interesting as they are molten salts at ambient temperature, which ensures better mixing. Moreover, they are non-flammable, have negligible vapor pressure and high chemical and thermal stability. *N*-alkyl-*N*-methylpyrrolidinium bis(trifluoromethanesulfonyl) imide (PYR_1A_TFSI, with 1 and A corresponding to the number of the carbon in the side chain, here N equals 3 to 5) were used as RTILs. PEO and LiTFSI were mixed and the viscous PYR_1A_TFSI was added. All the components were mixed prior an annealing step at 100 °C overnight and were hot-pressed for 2 min at 100 °C, resulting in a SPE [23].

Using a hot-pressing process is a rapid and easy way to shape SPE, which also has the advantage of being compatible with many different polymers as both temperature and pressure can be tuned to enable SPE shaping.

#### 2.1.3. 3D Printing

3D printing is a well-known method for the creation of objects from polymeric filaments. As SPEs are primarily comprised of polymers, 3D printing easily comes to mind as a method for producing them. Recently, Maurel et al. published a study describing the use of 3D printing to make a SPE along with the challenges that they encountered during this process. They used a 3D printer to fabricate a POE-based SPE with LiTFSI via fused deposition modelling (FDM). FDM is a technique where a polymeric filament is extruded and heated at the end of the nozzle to be deposited as a melted compound which solidifies after a few seconds of exposure to ambient temperature. This process must therefore be performed on thermoplastic materials. The heating temperature is selected to be a few degrees above the fusion temperature of the polymer. POE and LiTFSI were pre-mixed in a mortar and then introduced into an extruder in order to obtain a POE-LiTFSI filament with a diameter of 1.75 μm. The extruder and 3D printer were kept in a dry room to avoid any water contamination resulting from the hydrophilic nature of LiTFSI. The commercial 3D printer also needed to be modified as the introduction of LiTFSI to PEO plasticizes the polymer causing it to become stickier. The nozzle input and output were unchanged, with 1.75 mm and 0.4 mm diameters, respectively and the maximum Z-axis resolution was estimated to be around 200 μm. After the appropriate modifications to the 3D printer, the authors were able to obtain a 3D-printed SPE in a form of a 3 mm diameter disc. The conductivity of the sample was around 10^−7^ S.cm^−1^ at 30 °C which is rather low but expected for a PEO-based polymer. However, the conductivity at 90 °C was on the order of 10^−3^ S.cm^−1^, which is comparable to PEO-based SPE made by other processes [24].

This first demonstration 3D printed SPEs is very promising. The authors learned that using the technique to fabricate SPE is a challenging task. It requires the use of a polymer which is processable by the printer and has competitive ionic conductivity and electrochemical stability.

#### 2.1.4. Melt Processing

**Figure 3:**
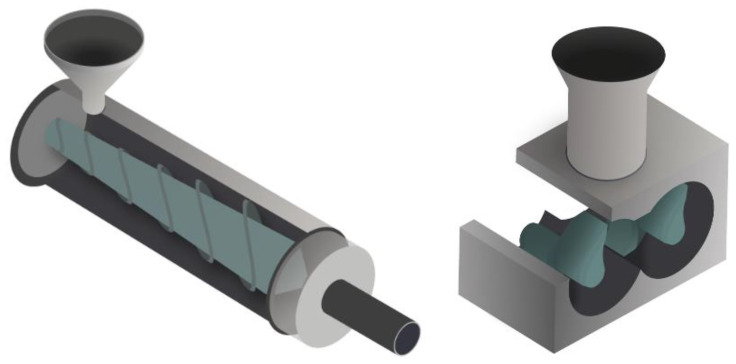
Extruder (**left**) and internal mixer (**right**) used to make solid polymer electrolytes. The polymer and the salt are often pre-mixed before their introduction into the chamber. Heating and high shear rates cause the polymer to flow allowing salt to be dissolved into it. This process is very interesting as it allows the polymer and the salt to be mixed in a few minutes as a result of high shear rates and the polymer being in its melt state.

The extrusion process, a well-known and wildly used process in the plastic industry, can also be used to make SPE. There has been interest in applying this process to battery manufacturing in both academic and industrial fields (Figure 3) [25,26,27,28]. However, industrial-grade extruders require high quantities of materials, which is often challenging at the academic scale. For this reason, the internal mixer is frequently chosen by academics as it fulfills the same purpose as the extruder.

Gray et al. [25] as well as U.S. patents issued by Raychem Limited [26] and Hydro-Quebec [27] focused on using extrusion with classical PEO. Although all these researchers used the extrusion process, several steps differed between the authors. Gray et al. first milled POE and LiClO_4_ in a ball-mill system and then introduced the mixture into an extruder heated at 150 °C under inert gas (we would like to advise readers that using any perchlorate salt in an extruder at high temperatures requires great care as perchlorates can explode if they crystalize in their acidic form). The cells were then directly filled with the extruded mixture and cooled under pressure in an argon-filled glove box [25]. Hydro-Quebec used the same approach for the extrusion, forming a film directly at the end of the extruder and covering the electrode with the SPE, thus increasing the surface contact at the interface between the solid electrolyte and the electrode [27]. Both these techniques are solvent-free. However, Cook et al. used highly polar liquid plasticizers, like ethylene carbonate (EC) or propylene carbonate (PC), to help processing the polymer-salt mixture and to help decrease the polymer crystallinity. With this approach, the preferred ratio of solvent is between 25 and 40 wt.%. The last step consists of hot-pressing the composite mixture in order to obtain a film [26]. In such a process, even though the amount of solvent used is less than in the solvent-casting method, the flammability and cost issues related to their use remain. As an alternative, Ma et al. used a similar approach but added glycerol to plasticize their blend and used water as the solvent. By doing so, the use of organic solvent was avoided. They formulated a SPE using starch as a matrix. They started by pre-mixing the starch with a solution composed of water, alkali metal salt (LiCl) and glycerol, which was used as a plasticizer. The mixture was stored for 24 h prior to extrusion, resulting in a SPE [28,29]. This attempt proves that extrusion is well suited for processing various polymers, not just PEO. However, the drying step must be monitored closely to ensure no water remains in the SPE. Recently, Caradant et al. used the melt-process to formulate polymeric blends to be used as solid polymer electrolytes. In this study, an internal mixer was used to blend Hydrogenated Nitrile Butadiene Rubber (HNBR) with PEO and LiTFSI. The process was as follows: first HNBR was introduced into the internal mixer and was processed alone at 170 °C for 4 min. The rotation speed was gradually increased from 5 to 30 rpm. PEO and LiTFSI were then added to the HNBR and the whole blend was mixed for 6 min at 50 rpm. Finally, the blend was pressed at 120 °C with a pressure of 5 bar to form 100–150 μm-thick films. This process allowed several SPEs with different HNBR:PEO ratios to be produced, demonstrating the versatility of the melt-process. The ionic conductivity of the resulting films was tested by impedance spectroscopy and the blend comprising 30 wt.% HNBR and 70 wt.% PEO, with 24.5 wt.% of LiTFSI, had a conductivity of 3.03 × 10^−5^ S·cm^−1^ at 30 °C, which is higher than that of 100 wt.% PEO with the same quantity of LiTFSI [30].

Solvent-free SPE can be processed by four methods: the direct dissolution of salt into the melted polymer or into a liquid low molecular weight polymer, the hot-pressing of a polymer-salt mixture, and the 3D printing of polymer filaments by FDM and extrusion. The latter is interesting because it is easily scalable and can be used on an industrial scale. However, extruding a SPE is non-trivial for several reasons such as having appropriate melt viscosity or a polymer melt temperature compatible with the lithium salt’s thermal stability. To tackle these challenges, the use of additives, like solvents or ILs, can be of interest. In this case, it needs to be mentioned that issues surrounding the solvent-casting method are still valid, however the amount of liquid to deal with should be much less constituting an improvement in the process. Secondly, polymers and lithium salt need to be heat-resistant and resistant to high shear rates, which needs to be taken into account when polymer blends are used in SPE.

In all-solid-state batteries, SPE are not the only solid-electrolytes that are considered as a replacement for liquid electrolytes. Hybrid polymer electrolytes have also raised significant attention. The solvent-free processing of hybrid polymer electrolytes will be detailed in the next section.

### 2.2. Hybrid Polymer Electrolytes

Hybrid polymer electrolytes are composed of a polymer, a lithium salt dispersed in the polymeric matrix, and fillers. Fillers are used to reach higher ionic conductivities than the ones achieved in traditional SPE. Two types of fillers can be used, ionically conductive and non-conductive. Non-conductive fillers, like silica or alumina, are added to the polymer-salt mixture to decrease the crystallinity of the polymer (large surface area and Lewis acid properties). When acting as solid plasticizers, the ionic conductivity is improved below the melting temperature of the polymer as the free volume is greater in the amorphous phase of the polymer [31]. Non-conductive fillers do not directly participate in the conduction of Li ions. Conductive fillers, which are solid lithium-containing ceramics known for having a lithium-ion transference number of 1, are expected to be involved in ionic conduction. Introducing particles into a polymer matrix raises new concerns regarding the dispersion of these particles and the homogeneity of the resulting composite. If one of these parameters is not optimal the ionic conductivity of the electrolyte will be impacted. Thus, the use of these two types of fillers, i.e., non-conductive and conductive, relating to the manufacture of solvent-free hybrid polymer electrolytes will be discussed.

#### 2.2.1. Melt Intercalation

**Figure 4:**
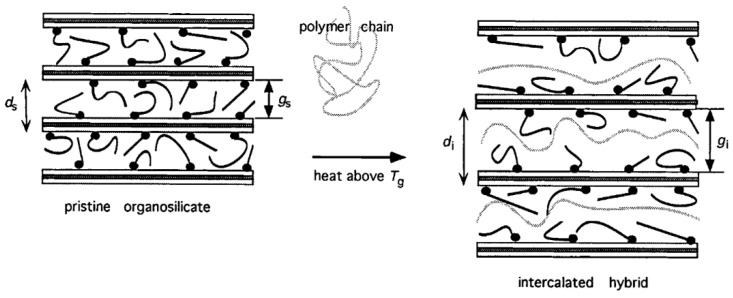
Melt intercalation of polymer chains into layered silicate. The temperature used to melt the polymer is its T_g_ if the polymer is amorphous or its T_m_ if it is semi-crystalline. g is the spacing between silicate layers (g_s_ < g_i_) and d is the silicate inter-layer distance. Reprinted with permission of Journal of Polymer Science Part B: Polymer Physics 1996, 34 (8), 1443–1449 [35].

In 1995, Vaia et al. reported the melt intercalation of a polymer into layered inorganics (silicate), without the use of any solvent. This process consists of mixing the polymer and the inorganic particles at ambient temperature. The polymer–inorganic filler mixture is then heated to the appropriate melt processing temperature. This step results in the intercalation of the polymer into the layered inorganic (Figure 4). The expansion of the inorganic inter-layer leaves space for the polymer chains, resulting is a stable composite. The authors used this process for the incorporation of POE into a Na^+^ or Li^+^-montmorillonite, creating a composite polymer electrolyte. As PEO is a semi-crystalline polymer, the composite layered electrolyte is heated at 80 °C (above PEO’s T_m_). By doing so, the authors were able to obtain higher ionic conductivities below 60 °C when compared to the SPE that is formed with PEO and LiClO_4_ [32]. The same process was used by Chen et al. to make a PEO-poly(methylmethacrylate) (PMMA) blend with a Li^+^-montmorillonite electrolyte [33]. Using layered silicate can be interesting because a lot of polymers have been studied with these fillers for other applications, like poly(dimethylsiloxane) (PDMS), and could be tested in the Li-ion battery context [34]. However, melting polymers into fillers is restricted to this particular type of filler, forcing other processes to emerge in order to incorporate other types of particles into hybrid polymer electrolytes via solvent-free processing methods.

#### 2.2.2. Hot-Pressing

The hot-pressing process that has already been described for SPE can also be used for hybrid polymer electrolytes as it is based on the melting properties of the polymer matrix. Several authors used this technique to shape composite electrolytes with different types of fillers. Appetecchi et al. used a ball-mill to mix PEO, LiCF_3_SO_3_ and a filler (fumed SiO_2_ or Al_2_O_3_) and pressed the mixture between 70–80 °C for 10 to 20 min at 3 to 4 tons [36]. Even though PEO is the most commonly used polymer, the pressing step parameters can be varied. It is possible to increase the temperature, the pressing time and the applied pressure to shape the hybrid polymer depending on the filler used [37,38]. This process is often used due to its efficiency. However, it is quite difficult to control the morphology of a composite made by hot-pressing.

Other melt-processing techniques, which generally offer better homogeneity of hybrid polymer electrolytes will be discussed in the next section.

#### 2.2.3. Melt Processing

To overcome the mixing limitations of the hot-pressing method, melt processing can be used as the morphology of the composite polymer electrolyte can be tuned using high shear rates, various temperatures or different screw designs [39]. It also presents the possibility of processing hybrid polymer electrolytes with non-conductive and/or conductive fillers, which is an asset of the process. The melt processing can be done with two distinct apparatuses: the extruder or the internal mixer, both of which work similarly.

##### Non-Conductive Fillers

One of the first attempts to extrude a hybrid polymer electrolyte was made by Hydro-Québec. To do so, they introduced nanoparticles of metal oxides like titanium oxide, fumed silica or aluminum into SPEs composed of a polymer and a salt. The goal of this patent was to demonstrate how the extrusion of SPEs can be finely tuned when the polymer and salt mixture tends to become sticky, making the extrusion process difficult. The process proposed in this patent is as follows: the polymer was mixed with a part of the fumed silica (for example), while another portion of the silica was mixed with the salt. These two mixtures were then combined to form a single blend to which the last portion of fumed silica was added. The last step was the extrusion through a flat die which resulted in the formation of a hybrid polymer electrolyte. The amount of fillers added is related to the salt concentration as well as the adhesive behavior of the polymer used as a matrix [40].

**Figure 5:**
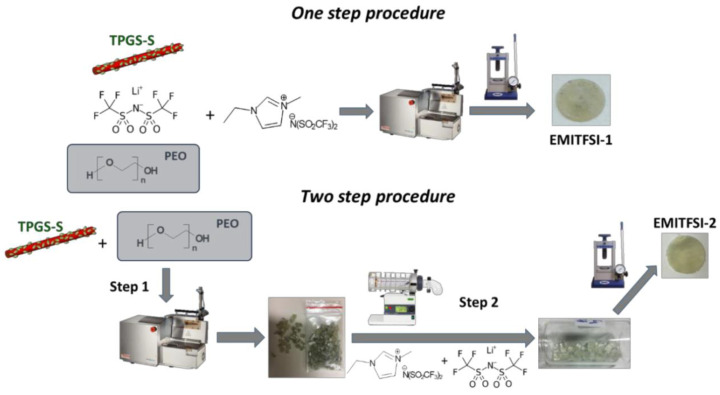
Extrusion processes used by Tiemblo’s group for hybrid polymer electrolytes of poly (ethylene oxide) (PEO), EMITFSI (or PYR_14_TFSI), LiTFSI, and surface-modified sepiolite (TPGS-S). The one step procedure consists of mixing and extruding all the components together while the two-step procedure begins with the extrusion and pelletization of PEO and sepiolite which is followed by swelling the pellets in a liquid mixture of EMITFSI + LiTFSI. Reprinted with permission of Polymers 2019, 11, 406 [49].

Another example of the extrusion process applied to the preparation of a hybrid electrolyte was reported in 2005 by Loyens et al., who produced a composite solid polymer electrolyte made of POE, silica and a sodium salt, NaClO_4_. They used a two-step melt compounding process. A master batch of PEO–silica was made in an internal mixer (120 °C, 40 rpm during 8 min) and was then placed into a twin-screw extruder, in which more PEO and salt were added to the master batch (120 °C, 80 rpm during 10 min). Finally, the electrolyte was shaped by compression molding in a 1 mm thick mold at 120 °C [41]. Following this attempt, it appears that extrusion with Li salt was not performed until ten years later by Tiemblo’s group, who has since become very active in improving their extrusion process [12,42,43,44,45,46,47,48,49]. In 2014, Mejia et al. physically pre-mixed PEO, LiTf, sepiolite (surface modified or not) and EC or a mixture of EC and PC. Surface modified sepiolite was added to the composite to act as a physical cross-linker, while EC and PC were used as plasticizers. All the compounds were then processed by extrusion at 80 rpm for 25 min at 120 °C and shaped by compression molding [42,43,44]. As previously discussed in Loyens’ study, it is worth mentioning that extrusion is a quick process. Since the first publication from Tiemblo’s group on melt processed solid electrolytes, some improvements, especially the suppressing of carbonated solvents, have been made in the extrusion process. Instead of using EC or PC as a plasticizer, ionic liquids, like 1-butyl-1-methylpyrrolidinium bis(trifluoromethanesulfonyl)imide (PYR_14_TFSI), have been employed [45,46,47]. The procedure is similar to the one described above and is schematized as the one step method in Figure 4. Replacing EC and PC by an IL was successful as the ionic conductivities of the composite electrolytes are on the same order of magnitude of 7.8, 7.0 and 3.3 × 10^−4^ for EC, EC:PC (1:1) [43] and PYR_14_TFSI [45], respectively (formulation is 34 wt.% PEO, 13 wt.% LiTf, 5 wt.% surface-modified sepiolite and 38 wt.% of plasticizer). However, the hybrid polymer electrolytes containing PEO, sepiolite and IL developed by Tiemblo’s group, were found to be in a solid-like state up to 90 °C, being liquid above this temperature. Even though the aim of the application is to obtain a functional hybrid electrolyte at ambient temperature, it is still an issue if the polymer electrolyte is known to flow at rather low temperatures. In order to obtain more resistant hybrid polymer electrolytes, the one step extrusion process (Figure 5) was used to formulate a poly (vinylidene fluoride-co-hexafluoropropylene) (PVdF-HFP) based solid electrolyte, using the same composition as previously detailed. Hence PVdF-HFP, 1-propyl-1-methylpyrrolidinium bis(trifluoromethanesulfonyl)imide (PYR_13_TFSI), surface-modified sepiolite and LiTFSI were successfully melt processed by extrusion [48].

The previous examples show that there is not just one extrusion method to formulate a hybrid polymer electrolyte, it is a tunable and versatile technique. Miguel et al. demonstrated this by modifying the above mentioned one step process to produce a two-step process and compared the solid electrolytes obtained by each method. The two-step process is depicted in Figure 5 and proceeds as follows: PEO and surface-modified sepiolite were mixed, extruded (120 °C, 80 rpm, 20 s residence time), and pelletized at the end of the extruder. The pellets were then melted in an oven at 80 °C, meanwhile a homogenous solution of 1-ethyl-3-methylimidazolium bis(trifluoromethanesulfonyl) imide (EMITFSI) and LiTFSI was prepared and poured over the pellets. The blend was left in the oven overnight to allow for the full swelling of the pellets. Finally, the pellets were compressed to form a film. This process resulted in amorphous PEO and a solid-polymer electrolyte above 90 °C. However, the ionic conductivity at 25 °C is of the same order of magnitude, i.e., 10^−4^ S.cm^−1^, as the one made with the one step procedure [49]. Moreover, the two-step procedure seems difficult to apply on an industrial scale. Considering this study, it seems that many parameters still need to be optimized to reach the best compromise between mechanical properties, ionic conductivity and scalability.

We can see that the melting process is becoming increasingly popular and that authors tend to have their own extrusion process for the production of hybrid polymer electrolytes with non-conductive fillers. A summary of different hybrid polymer electrolytes made by extrusion is presented in Table 1. The extrusion process is sensitive to particle loading, or more precisely it depends on the capacity of the polymer matrix to incorporate these particles, which can be limiting to some extent. However, it is not sensitive to particle type, meaning that this process can also be used to incorporate ionically conductive ceramics, which will be discussed in the following part.

##### Ionic Conductive Ceramics

Conductive fillers can contribute to the ionic conductivity of the hybrid polymer electrolyte as they contain lithium. As their microstructure is similar to the non-conducting fillers discussed above, these conductive fillers are compatible with the melt process.

Tiemblo’s group attempted to replace the non-conductive sepiolite with conductive garnet-type Li_7_La_3_Zr_1.75_Nb_0.25_O_12_ (LLZNO) using extrusion. The replacement of sepiolite was possible but due to the chosen formulation the resulting mixture was still viscous after processing. Since they were previously able to form solid composites with sepiolite, the choice was made to add some sepiolite to improve the mechanical strength of the film, which resulted in a solid electrolyte. In addition to the improved mechanical properties, the ionic conductivity of the hybrid sepiolite-LLZNO polymer electrolyte was similar to the viscous LLZNO-polymer electrolyte, around 10^−4^ S.cm^−1^ at 30 °C [12]. Huang et al. also made a hybrid polymer electrolyte with a garnet-type filler, Li_6.4_La_3_Zr_1.4_Ta_0.6_O_12_ (LLZTO). With their approach, no solvent was needed to melt process the PEO–LiTFSI–LLZTO composite. The process is as follows: PEO and LiTFSI were pre-mixed, then the mixture was placed in an internal mixer and heated to 150 °C. Once the PEO was melted, LLZTO was added. After homogenous dispersion, the blend was pressed to form films. Electrolytes with the same composition were made using the solvent-casting method and were compared. Huang et al. compared the homogeneity of the two blends by scanning electron microscopy (SEM) and stated that little to no agglomeration was present in the melt processed sample while significant agglomeration occurred in the sample that was processed via the solvent-casting method. By infrared spectroscopy, the authors detected the presence of a peak at 870 cm^−1^ which was ascribed to the asymmetric vibration of CO_3_^2−^ coming from the formation of LiCO_3_ when LLZTO is in contact with residual water or CO_2_ [51]. This peak was present in both cases, but they concluded that it was more intense when the hybrid electrolyte was made by solvent-casting demonstrating another advantage of solvent-free processing. Despite the encouraging homogenous morphology of the melt processed composite electrolyte, both blends had the same conductivity: 1.3 × 10^−5^ S.cm^−1^ at 25 °C [52]. It needs to be highlighted that 150 °C is a relatively high temperature for processing PEO and that chain breakage is expected to take place, which could explain the lower than expected conductivity for the melt processed hybrid electrolyte [53].

These examples, with inert and active ceramics, show that the melt process can be used to make hybrid polymer electrolytes and that this technique produces polymer composites with interesting properties and conductivities. However, as for all processes, there are some limitations regarding melt processing which will be further discussed.

##### Extrusion Parameters

The above-mentioned melt processed formulations show the versatility of the extrusion process. Hence, various polymers and fillers can be processed by extrusion, resulting in different mechanical properties and ionic conductivities. However, an aspect that needs to be considered is the impact of the extrusion process itself.

**Figure 6:**
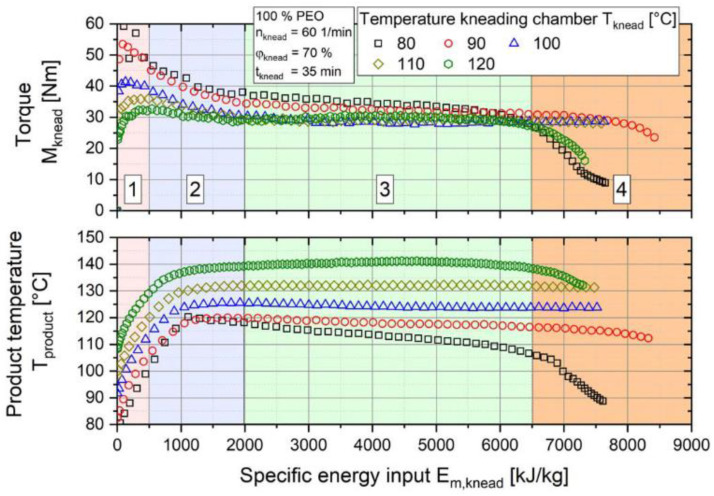
Influence of the processing temperature (T_knead_) on the polymer PEO. The composite undergoes four steps: (1) Homogenization, (2) Plasticization, (3) Plasticized homogenization and (4) Degradation. Reprinted with permission of Journal of The Electrochemical Society, 2020, 167, 020558 [50].

To have a better understanding of the extrusion process, Froboese et al. studied the impact of shear rate, temperature, time of the extrusion as well as the percentage of salt and filler used in a hybrid polymer electrolyte composed of PEO, LiTFSI and SiO_2_. The process is based on the extrusion of the components and does not require any liquid (organic solvent or ILs). At first, POE and SiO_2_ were mixed at 20 °C in a three-dimensional rotating mixer. LiTFSI was then added and all the components are granulated. The pellets were melt processed in an internal mixer at 100 °C and the resulting composite was calendered to obtain films. Knowing that the main component of these electrolytes is the polymer, the authors emphasized following the torque and temperature of PEO inside the internal mixer. Torque and temperature are two important process parameters to follow as the first indicates the physical resistance of the composite to the forced movement and can be translated into the quality of the mix while the second allows thermal decomposition to be avoided as temperature can rise quickly through shear. For example, the impact of the processing temperature is analyzed in Figure 6, where the melt passes through four phases. Phase 1 is the homogenization where the polymer particles are mixed, heated and partially melted. Phases 2 and 3 correspond to the evolution of the polymer plasticization reaching the stage of completely melted particles. Phase 4 represents mechanical and thermal degradation where polymer chains are damaged and is characterized by a decrease in the torque or the product temperature, respectively. The authors tested five processing temperatures 80, 90, 100, 110, and 120 °C and recorded the torque and product temperature (real temperature of the composite inside the chamber). They found that choosing the appropriate temperature (100 °C) avoids any degradation of PEO, which is a key parameter for any polymer electrolyte [50]. This aspect was also studied by Malik et al. who analyzed the behavior of a PEO/PVdF-HFP blend. They observed the degradation mechanism of this blend (without salt) that can be caused by the extrusion process. They pointed out that making a blend is fairly easy in theory but the two (or more) polymers that compose the blend should be processable in a similar temperature range. The degradation of PEO is known to happen above 135 °C, while PVdF-HFP needs to be processed in this temperature range. Size exclusion chromatography measurements revealed that the M_w_ of PEO decreases with the processing time, showing some degradation of the polymeric chains. By-products containing carbonyl or ester functions were identified by FTIR, gas chromatography and mass spectrometry confirming the degradation of PEO and proving that special attention needs to be taken when choosing polymers for processing by extrusion [53].

**Figure 7:**
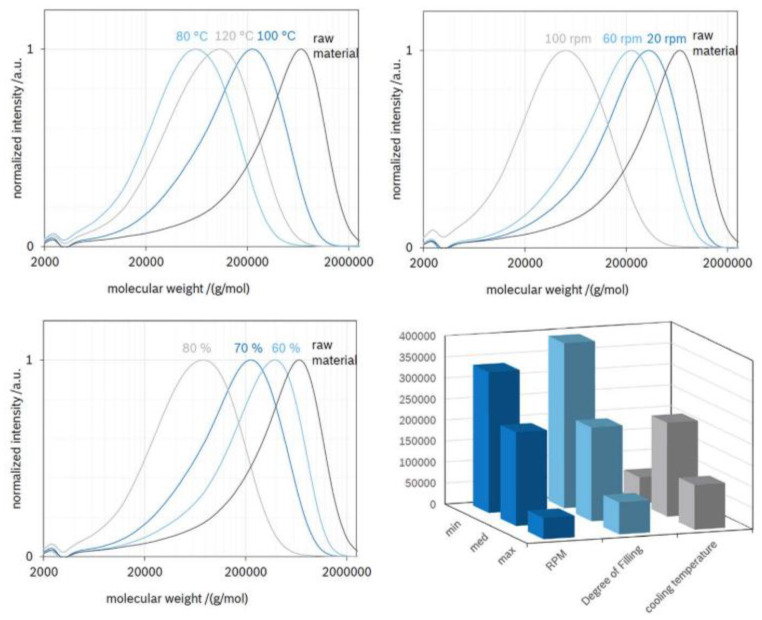
Impact of temperature (top left), shear rate (top right) and filler content (bottom left) during extrusion process of PEO. The chain lengths of PEO are analyzed by GPC and a summary of the impact of three parameters mentioned is made in bottom right corner. Reprinted with the permission of Journal of The Electrochemical Society, 2020, 167, 020558 [50].

Malik et al. pointed out the effect of temperature on the degradation of a SPE PEO-based blend [53], but Froboese et al. went further as they demonstrated that shear forces and filler content also have an impact on the resultant hybrid polymer electrolyte. Gel permeation chromatography (GPC) measurements on PEO show the chain lengths after different processing parameters and are reported in Figure 7. It reveals that increasing the shear rate as well as the filler content leads to a decrease in PEO molecular weight. This shows that all cited parameters impact the resulting composite and compromises need to be made. The optimal parameters window is very narrow, and polymer degradation should always be considered when the extrusion process is used. Moreover, the results presented for PEO with the tested parameters, will obviously be different for each potential polymer used as a matrix.

The extrusion process is a powerful method to make hybrid polymer electrolytes. It has the advantage of being fast and can ensure a high degree of dispersion and homogeneity of the composite. Nevertheless, the temperature and shear rate imposed during the mixing must be carefully considered as they impact the polymer matrix that is used.

Solvent-free hybrid polymer electrolytes can be processed by three methods: the melt intercalation of a polymer into layered silicates, the hot-pressing of a polymer-salt-filler mixture, and extrusion. Extrusion is the most relevant technique for the processing of hybrid polymer electrolytes as it tends to result in good particle dispersion which produces more homogenous composite materials with high shear rates.

The fact that dry processing can be used to make solid polymer-based electrolytes has been widely discussed. However, electrodes are another high loading component that is found inside batteries. Different solvent-free processes for the formulation of electrodes will be reported in the next part of this review.

## 3. Dry Processes for Electrodes

Electrodes for Li-ion batteries are commercially produced by dissolving the polymeric binder into N-Methyl-2-Pyrrolidone (NMP) and adding the active material and conductive fillers into the solution. After proper mixing, the electrode is casted onto a current collector using the doctor blade method. However, NMP is a toxic and costly solvent that needs to be replaced. The first alternative solvents that have come to light to replace NMP are water and ethanol [54]. These alternatives have been proposed for the purpose of making electrode processing a greener process. However, it still relies on solvents which can impact battery performances, especially water which needs to be absent in Li-ion batteries. Recently, a novel approach consisting of solvent-free techniques that has been proposed to confront the challenge of polymers being hardly soluble in solvents that are less toxic than NMP has been gaining popularity. Currently, three solvent-free processes are used to formulate electrodes for all-solid-state and conventional batteries. These techniques are based on melt processing and spraying.

### 3.1. Hot and Ambient Temperature Pressing

A hot-pressing process similar to the one described for both SPE and hybrid polymer electrolytes, has been reported for the formulation of non-porous electrodes. In 2007, Kim et al. reported the fabrication of a composite cathode for an all-solid-state battery. LiFePO_4_ (LFP) and conductive carbon were mixed together while PEO and LiTFSI were dissolved in PYR_1A_TFSI (A = 3 to 5). The powders were then added to the paste-like mixture to form a blend. The final step was to anneal the blend at 100 °C overnight and then hot-press it to form 1 mm-thick strips of composite cathode. Finally, the stripes were cold-pressed to reach an electrode thickness of around 50 μm [23]. In 2019, Kirsk et al. used the compressed molding process to fabricate porous electrodes. In their study, the electrode was formulated using only active material and conductive carbon, therefore producing binder-free electrodes. This is often challenging as this kind of electrode tends to have poor mechanical properties. A potential downside of this process is that it requires the use of compressible materials. LFP, a common electrode material, is not compressible so the authors chose to use holey graphene (hG), as the electronic conductive compound inside the electrode, which is known to be compressible. In order to produce homogenous electrodes, powders such as LFP and hG were pre-mixed together in a vortex mixer. The powder blend was then loaded into the stainless-steel pressing die and cold pressed for 10 min onto the current collector which can easily be peeled-off after the pressing step. Highs pressures, between 20 and 500 MPa, are necessary to obtain free-standing electrodes. One advantage of this process is that the thickness or porosity can easily be controlled by varying the applied pressure during the compression step. Also, energy dispersive x-ray spectroscopy (EDS) was used to prove that homogenous LFP:hG electrodes can be obtained via this process. Additionally, compressed molding was used to produce electrodes comprised of different active materials including LiNi_1/3_Co_1/3_Mn_1/3_O_2_ (NMC 111), Li_4_Ti_5_O_12_ (LTO), LiCoO_2_ (LCO) and LiMn_2_O_4_ (LMO). LFP:hG electrodes were further characterized and electrochemically tested. Their study shows that the electrochemical properties of the resultant electrode depend on the pressure that was applied during the compression stage. For example, LFP electrodes with a specific capacity of almost 150 mAh.g^−1^ could be maintained at 0.2 C after 50 cycles and 200 cycles, when pressures of 500 MPa and 20 MPa were applied respectively [55]. Both these results are promising but they raise concern regarding the ideal pressure that needs to be applied to obtain the best electrochemical performance from a given electrode formulation.

A similar process is often used to process non-porous electrodes made with solid electrolytes Li_2_S-P_2_S_5_ [56,57,58,59] and Li_6_PS_5_Cl [60,61,62]. For example, Auvergniot et al. formulated non-porous positive electrodes with LCO, NMC 111 and LMO active materials. Carbon fillers, Li_6_PS_5_Cl and the desired active material were grinded in a mortar and then pressed at ambient temperature with a pressure of 6.4 tons. The resulting electrodes were approximately 90 μm-thick. Another example was given by Hippauf et al. who used the viscosity of a melt polymer to properly mix the different components and connect the particles together. In their alternative method, NMC coated with Li_2_O-ZrO_2_ (LZO) was used as the active material. LZO-coated NMC, conductive carbon and the solid electrolyte Li_6_PS_5_Cl were mixed in a mortar at ambient temperature. The polymeric binder, poly(tetrafluoroethylene) (PTFE), was heated and added to the mortar. Here, PTFE was chosen for its ability to form fibers which results in linking the particles to one another without covering them. A highly viscous composite blend was obtained and processed by an external mixer to create a self-standing electrode with the appropriate thickness [62].

Using a press to produce electrodes made from Li_6_PS_5_Cl or Li_2_S-P_2_S_5_ is a fast and easy process as these materials can be pressed at ambient temperature. However, it should be mentioned that this procedure tends to yield highly heterogeneous electrodes due to the high loading and variety of particles. For this reason, other protocols are studied.

### 3.2. Spray Deposition

Another alternative method to wet processing has been developed by the company Maxwell Technologies [63] and has subsequently been used by several research groups. This process is based on Electrostatic Spray Deposition (ESD) [64,65,66,67,68,69]. During the process, the particles, i.e., the active material, the conductive carbon and the binder (which is in powder form) are mixed together and are vaporized onto a metallic current collector due to the application of a high voltage. The last step consists of calendering the electrode to obtain the desired thickness and control the porosity. In some cases, an extra heating step is added in order to melt the polymer binder to ensure cohesion between particles. Another alternative method was discussed by Parl et al. who used a similar process, though instead of applying ESD, they worked with a highly pressurized argon gas flow to deposit the particles onto the current collector [70].

Spray deposition techniques are interesting as they could be widely used for all types of particles. However, this process still has some limitations regarding control over the thickness of the electrodes. Even if electrode thickness can be tuned by pressing, it is highly dependent on the initial deposition where homogenous thickness is difficult to achieve. The extrusion process, which is discussed in the next section, can be used to overcome this challenge.

### 3.3. 3D Printing for Electrodes

In the literature, two 3D printing techniques have been reported for the manufacture of electrodes. The first technique is called liquid deposition modelling (LDM) [71,72] and, as the name suggests, it is a solvent-based method. For this reason, it will not be further discussed in this review as we are focusing on dry processes. The other method, which is of interest for us, is FDM as was described in the SPEs section above. Maurel et al. published a review which provides more details and examples regarding the use of these two techniques to produce electrodes for lithium batteries [73].

FDM was first used to prepare electrodes by Foster et al. in 2017. They used a commercial graphene-based polylactic acid filament (graphene/poly(lactic acid) (PLA)) containing only 8% graphene and 92% PLA and obtained a free-standing anode. As expected, the electrochemical performances were poor due to the low percentage of graphene. However, this study provided proof-of-concept that FDM could be used to make electrodes for Li-ion batteries [74]. In 2018, Ragones et al. developed PLA/LTO anode and PLA/LFP cathode composite filaments. The filaments were composed of 50 to 70% active material, 10% carbon additives, and 20 to 40% PLA. They dissolved PLA pellets into 1,3-dioxolane and added the active materials (LFP for cathodes or LTO for anodes), graphite, graphitized multiwalled functionalized carbon nanotubes (surface modified MWCNT) and Carbon black (CB). After solvent evaporation, the blend was crushed to obtain pellets which could be directly fed into the extruder in order to make a composite filament. With a nozzle diameter of 1.4–1.7 mm and a nozzle temperature between 190 and 210 °C, filaments with a diameter of 1.75 mm were produced. Finally, the electrodes were tested in the half cell configuration, showing partial utilization of about 50% of the theoretical capacity of the LFP cathode and about 60% of the theoretical capacity of the LTO anode [75]. In 2018, Reyes et al. also developed LTO anodes but used LMO to produce cathodes. Their formulation allowed for up to 30 vol.% of solid content into PLA, with an optimized ratio of active material to conductive carbons of 20:80. Unfortunately, this ratio is still very far from classical electrode formulations where high active material loading is needed (around 90 wt.%). The electrodes were made by first dissolving PLA into dichloromethane and then adding either CB, graphene, or MWCNT. After extrusion of the filament and 3D printing, anode and cathode disk electrodes with a thickness of 150 μm and a diameter of 14 mm were obtained. The most promising formulations were 6/24/70 vol.% for LTO/graphene/PLA and 4/16/80 vol.% for LMO/MWCNT/PLA [76]. Due to the very low quantity of active material (AM), the electrochemical performances of these electrodes were poor compared to electrodes prepared via classical techniques. The main limitation of these previous studies is the extremely low AM content of their electrodes. This problem was tackled by Maurel et al. who made highly loaded graphite/PLA electrodes. To do so, they added liquid plasticizers such as PC, poly(ethylene glycol) dimethyl ether (PEGDME) and acetyl tributyl citrate. The PLA/Graphite ratio was kept constant, 30/70 wt.%, and various percentages of additives, from 0 to 60 wt.%, were tested to determine the most efficient formulation. The overall process to prepare the polymer filaments was the same as previously described. After optimization, the most promising electrode was 32.8/49.2/13.1/4.9 (PLA/Graphite/PEGDME/CB in weight percent). The reversible capacities for solvent-casted films obtained over five cycles at C/20, C/10, and C/5 are promising with values of 342, 333, and 322 mAh.g^−1^, respectively. Unfortunately, the 3D printed electrode is not yet as good as the wet electrode due to limitations in thickness induced by the 3D-printer [77]. This clearly shows that decreasing electrode thickness is the next challenge to overcome in the development of this process. In 2019, Maurel et al. used a similar approach to produce the most efficient highly loaded cathode with LFP. The best formulation was the same as that which was found for the graphite anode: 33/49/13/5 (PLA/LFP/PEGDME/CB in weight percent). However, the capacities are still very low: 83 mAh.g^−1^ at C/20 and 43 mAh.g^−1^ at C/10. The electrode thickness was limited to 200 microns due to the low thickness resolution of the 3D printer [78].

The advantage of using 3D-printed electrodes is that the shape of the electrode can be controlled. However, even with the FDM process, solvent is still required during the first step to dissolve PLA and then to mix all the components together. This process could be improved by developing a method that would enable all the components to be put directly into the extruder and mixed without the use of a solvent. During the past few years, efforts have been made to increase the active material proportion of the filament. However, when compared to classical formulations that are used in wet processing and those used in other dry processes like direct extrusion and calendaring, there is still room for improvement. It is probably only a matter of time before this technique is efficient enough to produce competitive batteries.

### 3.4. Melt Processing

The last method to create electrodes that will be discussed here is the extrusion process. As previously mentioned in the sections describing the fabrication of SPE and hybrid polymer electrolytes, this process is very interesting as it is fast and easily scalable. However, forming an electrode with such a process is even more challenging as the polymer content is often very low and particle loading is high. These two parameters are the opposite of what is typically used in the classic extrusion process, which works well for mixtures with high polymer content and low to medium filler content. To solve these problems, researchers have been creative by using solvents or various additives to ensure processability. In the following sections, various electrode processing attempts by extrusion will be reported to present a global review of the literature. It is expected that analyzing past trials could help enhance protocol in future studies. Following the same trend, we will also report on the extrusion processes to create non-porous and porous electrodes.

#### 3.4.1. Non-Porous Electrodes

For all-solid-state batteries, no porosity is required in the electrode and the void is often filled by adding more polymer and salt. Doing so makes it easier to formulate an electrode using extrusion. Significant developments in the field of extrusion-processed electrodes were made in industry at the end of the 1990′s and the beginning of the 2000′s.

In 1994, Valence Technology patented the use of an extruder to formulate a cathode material. However, no further details were given regarding the formulation that was used in this device. It was only stated that the shear thinning cathode was deposited onto a current collector after going through a nozzle at the end of the extruder. An extruded electrolyte and an anode can then be directly deposited on top of each other to form an all-solid-state battery [79]. Similarly, another example of electrode production by extrusion was given by Bolloré Technologies who filed a patent in 1997 presenting the use of the extrusion process to make non-porous electrodes assembled with SPE that was also made by extrusion. Cathodes made using this process are typically composed of MnO_2_, amorphous carbon, PEO and LiCF_3_SO_3_ while the SPE is made of PEO, polyolefin wax, and LiCF_3_SO_3_ [80]. No further details relating to the use of the extrusion process to make electrodes were presented except for a die that allowed the formation of an electrode sheet that can be platted directly onto a current collector. This extrusion process is now used industrially, and the resulting all-solid-state batteries have been used in commercial electric vehicles [81].

At the end of the 1990′s, two other patents, both from the company W. R. Grace & Co.-Conn., that also claimed to manufacture non-porous electrodes for an all-solid-state battery by a one-step extrusion process, were filed. The electrodes were composed of the active material, conductive carbon, a polymer (PVdF or PAN for example), lithium salt and a solvent that is able to dissolve the salt and plasticize the polymer, PC or PAN for example. As a solvent is needed, this attempt to use extrusion to create an electrode cannot properly be called a dry process however, it allows for a better understanding of the evolution of its use for electrode production. The polymer and the salt were introduced in the appropriate solvent and mixed together. The solid active material and the conductive carbon mixture were then added to the liquid blend. The whole blend was fed into an extruder for proper mixing. At the end of the extrusion process, the composite passed through a die to form an electrode sheet. This electrode sheet can be directly platted onto a current collector and be used as is [82,83]. However, the authors did not mention the extrusion conditions (temperature or shear rates), or how they proceeded to remove the solvent used at the beginning of the process.

In 2004, Lavoie et al. also proposed a similar process. Their patent was based on the formulation of positive electrodes composed of at least 50 wt.% of particles. The electrode was comprised of polyether, lithium salt, electronic conductor (graphite and carbon) and active material. The latter can be processed through a single or twin-screw extruder. PEO is first introduced into the extruder, followed by a mixture of active material, lithium salt and electronic conductive material. After proper mixing, the blend is extruded through a die to form an electrode sheet, which is deposited onto a current collector. With the aid of calendering or laminating, an electrode thickness between 35 and 125 microns was reported [84].

All-solid-state batteries have been successfully implanted into electric vehicles, which have driven more than a total of 300 million kilometers [81]. Additionally, extrusion can be used to produce porous electrodes. This process has attracted much attention. The next section focuses on the production of porous electrodes to provide a better understanding of how the extrusion process can be applied to electrode manufacturing.

#### 3.4.2. Porous Electrodes

Recently, several groups reported the use of this process to create porous Li-ion battery electrodes [85,86,87]. However, when it comes to creating a porous electrode, the polymer content must be very low, and the porosity must be high to allow the electrode to be used with liquid electrolytes. These features are very challenging to produce and might be responsible for the fact that the extrusion process has not been used to produce porous batteries until very recently.

Different steps and approaches have been used to melt process electrodes. The extrusion process was first used by Haarmaan et al. However, the solvents were not completely removed from their formulation. They based their study on work by Dreger et al. [88] who used 50 wt.% NMP in their process by lowering the NMP amount to 20 to 35 wt.%. The procedure used to formulate the electrodes is as follows: all powders (NMC, conductive carbon and PVdF) were pre-mixed in a turbula. The mixture was then introduced into the extruder while NMP was added. The use of NMP was necessary as PVdF is not a thermoplastic, hence not directly usable inside an extruder. Also, PVdF has a high melting temperature which makes it difficult to use in the extrusion process [85]. The latest report on this hybrid extrusion process requiring NMP was published by Seeba et al. Like Haarman et al., the authors needed to use the solvent because the binder that was used in their electrode’s formulation was PVdF. Their electrodes were made with NMC 622, CB, conductive graphite and PVdF in NMP. At first, all the powders were mixed together while a 13 wt.% solution of PVdF was prepared. Then, the solution and the powders were kneaded together until granules with a solid content of 85 wt.% where obtained. This is higher than the solid content in the electrodes that were proposed by Haarman et al. The previous compounds were introduced into the extruder for processing and the resulting paste was extruded through a slot-tie and directly coated onto the current collector. An extra step of polishing was required to obtain a homogeneous electrode thickness. Finally, the solvent was evaporated at 120 °C [89]. This process is a hybrid between the classical wet process, as NMP is required to dissolve PVdF and make it processable, and a melt process as an extruder is used.

**Figure 8:**
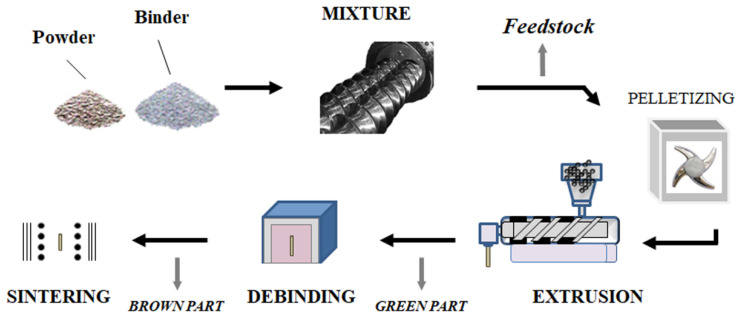
Fabrication process based on extrusion used by Sotomayor et al. Reprinted with permission of Journal of Power Sources 437, 2019, 226923 [86].

Another example is from Sotomayor et al. who used extrusion to formulate LTO and LFP electrodes without the use of any solvent. This process, which is depicted in Figure 8, comprises the following steps: particle mixing, extrusion, debinding and sintering. The classical binder, PVdF, is replaced by a blend of thermoplastic polymers which can be directly used in extrusion. The blend is composed of polypropylene (PP), paraffin wax and stearic acid (SA) [90]. Ceramic is also added to increase the ionic conductivity of the electrode. The active material, PP, paraffin wax, SA, and ceramic were introduced into the extruder. To maximize the homogeneity of the composite, this step was repeated three times and was then followed by the pelletization of the melt. The pellets were introduced into a single-screw extruder where they were mixed again. After this step, electrode sheets of various thicknesses can be obtained. The polymer blend (PP, paraffine wax, and SA) was then removed from the electrode by heating, which creates pores inside the electrode. A double heating step in n-heptane was required to degrade the polymers. Finally, electrodes were sintered at high temperatures in order to create cohesion between the remaining particles [86]. This study proves that porous electrodes can be made by solvent-free extrusion even if the particle content is high.

A similar process (Figure 8) was recently used by De la Torre-Gamarra et al. (2020) to make binder-free self-supported thick LFP-electrodes of approximately 500 microns [91]. In this study, the sacrificial binder (PP, paraffin wax and SA) and the powders (LFP and conductive carbons) were first mixed at 180 °C for 40 min. Then, the pellets were introduced into the extruder where the temperature profile of the barrel was optimized at 175/178/182/185 °C. For further electrode improvement, the impact of formulation on electrode performance was studied. The binder/powders ratio was varied (volume ratios: 50/50, 49/51, 47.5/52.5, 45/55, 43.5/56.5, and 42/58) and the resulting viscosity of the blend was analyzed by rheology. The viscosity of these mixtures followed an Arrhenius-type law:

η = η_0_ e^Ea/RT^

where E_a_ is the flow activation energy, η_0_ is the viscosity at a reference temperature T, and R is the ideal gas constant. The authors found that the highest powder content led to the highest flow activation energy. This parameter is important as it is directly related to the behavior of the blend as it exits the extruder. A high flow activation energy will ensure good retention of the geometry of the composite electrode after it is pushed out of the nozzle as the temperature decrease will provoke an increase in the viscosity. The optimal electrode formulation contained 55 wt.% powder. Once the appropriate ratio was chosen, the electrode was first thermally treated in n-heptane to decompose the polymeric phase and was then sintered to improve the mechanical strength of the final electrode. The sintering step was performed under inert N_2_ atmosphere to prevent the oxidation of LFP. The effect of the sintering temperature on the particles was investigated between 550 and 850 °C. At a higher temperature, a secondary LFP phase was produced, thus temperatures above 850 °C were eliminated from this experiment. The impact of the whole extrusion process on the electrochemical properties of LFP/conductive carbons was investigated. To do so, an electrode extruded and sintered at 600 °C was ground and compared to pristine LFP/conductive carbons particles. Electrodes containing 80 wt.% LFP, 7.5 wt.% of acetylene black, 7.5 wt.% of graphite and 5 wt.% of Teflon were prepared. This process did not alter the properties of these powders as a plateau was seen at 3.39 V and that the reversible capacity was of 162 mAh.g^−1^ at when cycling at C/20 in both cases. It was also shown that thick extruded electrodes are better impregnated with low viscosity electrolytes like PC:EC:DMC (1:1:1 in volume) + 1 MLiClO_4_ than when using only PC as solvent. This finding resulted in better cycling performances at moderate to high C-rates. This study confirms that it is possible to create porous electrodes without using any toxic solvents. However, as for electrodes prepared via solvent processing, it is necessary to investigate the parameters involved during electrode preparation to optimize the final product.

**Figure 9:**
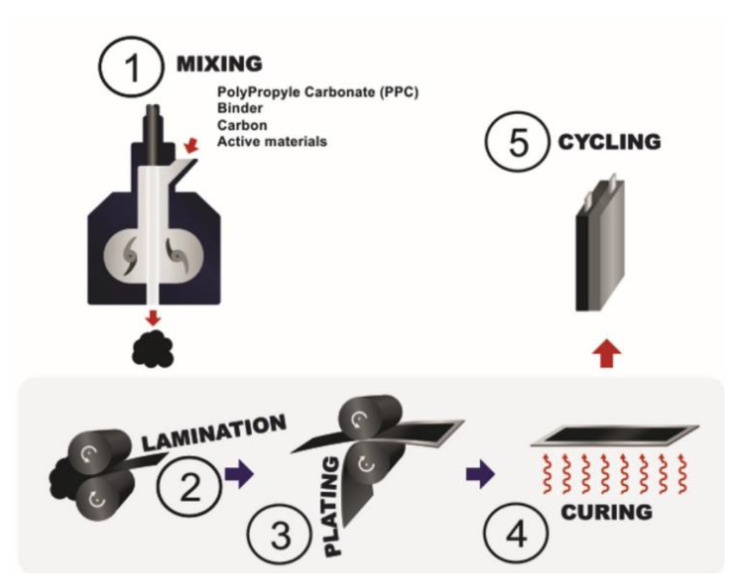
The different steps of the extrusion process developed by El Khakani et al. to formulate Li-ion battery electrodes. Reprinted with permission of Journal of Power Sources 454, 2020, 227884 [87].

The most recent use of solvent-free extrusion was reported by El Khakani et al. [87] and is based on Hutchinson’s patents [92,93]. In this case, an extruder or an internal mixer can be used and, like in Sotomayor et al. study, PVdF was replaced by an extrudable blend. The latter is composed of polypropylene carbonate (PPC) and hydrogenated nitrile butadiene rubber (HNBR). Figure 9 represents the different steps involved in the process. HNBR and PPC were first introduced into the internal mixer until a homogenous molten blend was obtained. The active material (LTO, LFP or NMC) and conductive carbon were then incorporated into the polymeric blend and mixed until they were properly dispersed. The composite mixture was laminated to form a sheet. This operation was repeated several times until the desired thickness was reached. The electrode was then plated onto a current collector. Finally, the electrode was heated to remove PPC, creating porosity inside the electrode. Astafyeva et al. used the same process to fabricate lithium nickel cobalt aluminum oxide (NCA) cathodes and graphite anodes. The type of binder used in their study is not directly mentioned in their work but they specify that elastomer or thermoplastic binders are used with conductive carbons and at least 90 wt.% of active material [94]. This technique is similar to the one proposed by Sotomayor et al. except that no solvent is needed at any stage of the process. Another aspect highlighted by these two studies is the control of porosity thanks to a polymeric sacrificial phase. It shows the advantage of melt-processing porous electrodes instead of using the classical wet method where porosity is dependent on the solvent.

The different extrusion processes show the feasibility and versatility of the procedure, which can be used to create porous or non-porous electrodes with various active materials. In addition, the melt process allows for better control of the electrode thickness compared to the solvent-based method.

The replacement of NMP in the electrode making process has been studied for over thirty years now. Exchanging NMP for other less toxic solvents was the first strategy that was attempted but it turned out to be very challenging as polymers are often insoluble in other solvents. Therefore, alternative methods for reducing/replacing NMP have been encouraged. Hot-pressing, ESD and extrusion are promising process for the next industrial generation of electrodes.

#### 3.4.3. Extrusion Parameters for Electrode Preparation

As previously mentioned in the solid electrolyte section, parameters like temperature and shear rates have a significant impact on the resultant polymer component. Dreger et al. specifically studied the impact of dispersing during extrusion and calendaring on the resulting electrodes [95]. In their paper, they made both anodes and cathodes. The anode was made with surface modified graphite, CB and PVdF while the cathode was made of NMC 111, CB, and PVdF. As in their previous publication, PVdF was dispersed in NMP. In the extruder, the final suspension was composed of at least 50 wt.% NMP for anodes and 40 or 65 wt.% for cathodes. For this reason, this process cannot be considered a dry process, but the method and the conclusions drawn from this article can still give a notion of the impact of screw speed rotation. The impact of extrusion time and temperature were not studied and were therefore kept constant. The extrusion time was between 5 and 7 min and the extrusion the temperature was 35 °C. The rotation speed was modified between 120 to 1200 min^−1^ as well as the conveying volumes. At similar screw rotational speeds, the viscosity of the blend can be correlated to the size of the carbon agglomerates. In this study, cathodes which are less viscous showed a narrower distribution of agglomerate sizes, less than 10 microns, compared to anodes which had agglomerates sizes up to 20 microns. Another observation was made by increasing stress input with higher screw speeds. In this configuration, larger agglomerates decomposed to form smaller agglomerates as expected. The reduction of agglomerates size was also associated with lower electrical resistance in the electrode which could be related to a better dispersion and thinner agglomerates leading to increased electrode homogeneity. On the other hand, decreasing the quantity of CB agglomerates leads to less porosity in the electrode as CB acts as a spacer between the AM. It is possible for smaller CB aggregates to exist between the AM or around its surface. This can lead to ionic diffusion path blocking which can be detrimental for the electrochemical performance of the battery. This shows that a careful compromise must be made with respect to the size of the CB agglomerates. In general, it shows that medium-sized CB agglomerates are preferred for the optimization of electrochemical properties. Dreger’s study shows that if the rotational speed is too slow, the resulting electrode will be inhomogeneous. But, if the rotational speed is too high, it will induce ionic diffusion path blocking due to small CB agglomerates. While the CB agglomerates can be broken by increasing the rotational speed, they can also be broken by increasing the processing time. The impact of extruder residence time on the electrode viscosity was studied by Sheeba et al. They showed that when increasing the number of passes from 1 to 3, the blend viscosity of the mixture containing 4 wt.% CB increased while the blend viscosity of the mixture containing 8 wt.% CB decreased [89]. They concluded that the increasing effect is related to the breakage of CB agglomerates leading to an increase of the surface area which diminishes the binder to surface area ratio resulting in a higher viscosity. The opposite trend was observed in the sample with 8 wt.% CB as it was believed that the surface area of the blend was already too high to see the impact of agglomerate breakage. These studies highlight the impact of screw rotational speed and processing time and how these parameters need to be tuned for electrode optimization.

The impact of the nature of the carbon particle was investigated by Sheeba et al. While keeping the binder content constant (here 3 wt.%) the authors investigated the nature of two types of carbon particles: CB and graphite, which is normally knows for its lubricating effect. It was shown that an increase in CB, from 4 to 8 wt.%, leads to higher torque. Replacing a part of the CB with graphite, i.e., having 4 wt.% of CB and 8 wt.% graphite, leads to an increase in torque compared to a mixture containing only 8 wt.% CB. This shows that, like in the classical wet process, the nature of particles impacts the viscosity of the blend. More precisely, the particle shape was found to play a key role. In this study, CBs were spherical with a diameter of 50 nm while the graphite particles are flake shaped and are much larger, i.e., 3 microns [89]. This corroborates the findings as small and spherical particles tend to move more easily than bigger flake-shaped particles thus decreasing the torque. This study demonstrates that using CB is more favorable for the process as the viscosity of the blend will be lower compared to using the same proportion of graphite.

As for solid polymer electrolyte, using the melt process is very promising to make both non-porous and porous electrodes. This process is particularly interesting as it could be used to form a whole Li-ion battery. However, the previous studies show that the extrusion process still need some improvements and optimization to take fully advantage of this process. The rotational speed, the processing time, the formulation, and the nature of AM or conductive carbons are various parameters that will directly impact the electrochemical performances of the electrode if not optimized. Yet, the electrode performances are very encouraging, and researchers only need to keep in mind that each of the previous parameters will probably need to be tuned for each formulation to make it more efficient.

## 4. Conclusions

Solvent-free processes to make solid and hybrid polymer electrolytes as well as electrodes, for Li-ion batteries, have been reviewed. Hot-pressing, 3D printing, and extrusion processes can be used for both electrodes and electrolytes. This is promising because it can allow one type of equipment to be used to formulate all battery components. Hot pressing and extrusion are also interesting because the processing time is very short compared to the classical solvent-casting method where several hours are often required to achieve proper homogeneity of the slurry, to dissolve the polymer and to evaporate solvents during the final drying step. Regarding 3D printing, the first step consisting in the dissolution of the polymer into solvent still needs to be removed from to process to lead to an efficient and more environmentally friendly process. Finally, another advantage of hot-pressing and extrusion techniques is their scalability for use in industry. Nevertheless, due to the high shear rates that are allowed by the extrusion process, electrodes and electrolytes are expected to be more homogenous than those that are made via the hot-press procedure. It also needs to be highlighted that despite their categorization as solvent-free processes, avoiding the use of solvents is a real challenge, as several authors still needed to use some solvent to extrude their composites. However, recent successes in solvent-free processing prove that is it possible to overcome this challenge. These examples open the door for the possibility of creating the non-porous composites which are required in all-solid-state batteries via dry processing methods.

For future developments, we would like to raise the attention of readers about the comparison between the classical process and new processes but also between new processes. So far, studies are focusing on showing the application of a new process, but no comparison can truly be made as various parameters are often different from one study to another. The formulation, the nature of the components, the characterizations (especially regarding cycling rate of a battery) are chosen specifically by each author, leading to difficult or even impossible comparison between research groups. From our point of view, this is a critical aspect that could be improved.

From this review, it appears that the polymer in electrode or electrolyte is a key component as many of the processes are derived from the polymer industry. Thus, two factors need to be clearly managed: the type of polymer that is used with a specific process and the loading content of particles. These two aspects are the ones which are limiting so far. Regarding the polymer, two paths can be considered. The first one is to change, chemically modify, or synthesize a polymer to fit the process while the second would be to suppress the polymer like in Li-S battery were the positive electrode is made by heating the sulfur followed by its penetration into mesoporous carbon matrix [96].

So far, the choice of a formulation can be narrowed thanks to Design of Experiments (DOE) but this is still a long process as several trials need to be made. With the development of machine learning and its use in the battery field, we think that this task could be greatly enhanced thanks to this approach [97]. The use of solvent-free processes seems to be limitless and even new composition of electrolyte or electrode could be developed, as long as the performances of Li-ion battery are ensured.

## Figures and Tables

**Table 1:** Composition of different hybrid polymer electrolytes containing non-conductive ceramics processed by extrusion.

Polymer	Salt	Filler	Additives	References
PEO	NaClO_4_	Silica	-	[41]
PEO	LiTFSI	Silica	-	[50]
PEO	LiTf	Surface modified sepiolite	EC or EC:PC	[42,43,44]
PEO	LiTf	Surface modified sepiolite	EMITFSI, EMIFSI, PMPFSI, PMPTFSI (PYR_13_TFSI), BMBFSI, BMBFSI (PYR_14_TFSI)	[45,46,47,49]
PVdF-HFP	LiTf	Surface modified sepiolite	PYR_13_TFSI	[48]

## Data Availability

Not applicable.
